# Bacteriophage Therapy in Companion and Farm Animals

**DOI:** 10.3390/antibiotics13040294

**Published:** 2024-03-23

**Authors:** Laura Bianchessi, Giulia De Bernardi, Martina Vigorelli, Paola Dall’Ara, Lauretta Turin

**Affiliations:** Department of Veterinary Medicine and Animal Sciences—DIVAS, Università degli Studi di Milano, 26900 Lodi, Italy; laura.bianchessi@unimi.it (L.B.); giulia.debernardi@studenti.unimi.it (G.D.B.); martina.vigorelli@studenti.unimi.it (M.V.); paola.dallara@unimi.it (P.D.)

**Keywords:** bacteria, bacteriophage, phage, therapy, challenge, alternative antimicrobials, veterinary, antibacterial, multidrug resistant

## Abstract

Bacteriophages, which are viruses with restricted tropism for bacteria, have been employed for over a century as antimicrobial agents; they have been largely abandoned in Western countries but are constantly used in Eastern European countries with the advent of antibiotics. In recent decades, the growing spread of multidrug-resistant bacteria, which pose a serious threat to worldwide public health, imposed an urgent demand for alternative therapeutic approaches to antibiotics in animal and human fields. Based on this requirement, numerous studies have been published on developing and testing bacteriophage-based therapy. Overall, the literature largely supports the potential of this perspective but also highlights the need for additional research as the current standards are inadequate to receive approval from regulatory authorities. This review aims to update and critically revise the current knowledge on the application of bacteriophages to treat bacterial-derived infectious diseases in animals in order to provide topical perspectives and innovative advances.

## 1. Introduction

Bacteriophages or phages (for short) are viruses that are parasitic to bacteria. They have been employed to treat bacterial infections since the beginning of the previous century, in the pre-antibiotic era, long before their nature was clarified; regulated clinical trials have only been performed and well documented in the literature in the modern era. Western countries partially abandoned their use after the large-scale deployment of antibiotics, but Eastern European countries have commonly maintained their use.

Clinically relevant issues linked to the rise of antibiotic resistance in bacteria have led to the necessity to urgently develop therapies as alternatives to antibiotics for the control of bacterial spread. In this context, there has been a renewed interest in bacteriophages, supported by a gradual increase in studies aimed at developing and testing phage-based strategies for combating multidrug-resistant bacteria.

In this paper, the recent developments of bacteriophage therapy are reviewed with the aims of critically analyzing the up-to-date progress and the current applications in the veterinary field, pointing out advantages and drawbacks, and showing the gaps that need to be filled to address research priorities.

## 2. Antibiotic Resistance

Soon after the discovery of the first antibiotic, penicillin, by Alexander Fleming in 1928, more antibiotics were discovered and subsequently produced by chemical synthesis, generating a beneficial revolution in healthcare and medicine. Fatal diseases could be effectively treated, surgery- and childbirth-linked risks tremendously diminished, and life expectancy was extended. Unfortunately, the widespread application of antibiotics to cure infectious diseases in humans and animals and for medical procedures, along with the irrespective overuse (or misuse) of antibiotics as animal growth promoters or disease preventives, led to the accumulation of antibiotic residues in the environment and the rise of antibiotic-resistant bacteria in humans and animals.

Antibiotic resistance, which is referred to as the inherited ability of bacteria to survive and reproduce where high concentrations of antibiotics are present, may either be natural or acquired [1]. The first kind (natural or intrinsic) is independent of the compound exposure and may be due to the natural lack of certain structures or the existence of antibiotic resistance genes (ARGs) in the bacterial genome for phenotypic resistance, owing to the hampered intake, inactivation, or outflow of antibiotics; acquired resistance can occur through the mutation or horizontal gene transfer of plasmid/chromosome portions and may be due to enzyme-catalyzed modification, which is the inactivation or outflow of antibiotics. However, the genetic determinants for this trait arise from the adaptation process of the microorganism to the presence of antibiotics (selective pressure) [2].

A recent study demonstrated that there are already over 2500 ARGs spread in all habitats; their abundance is correlated with high anthropogenic activities, and they are collectively responsible for resistance to 24 different classes of antibiotics [3]. The complex biochemical and physiological mechanisms underneath the emergence of antibiotic-resistant bacterial strains under selective pressure, primarily caused by the widespread use of antibiotics, are currently under investigation. This phenomenon is responsible for about 700,000 global deaths per year at present, with an estimated increase to above 10 million in 2050 if corrective measures are not implemented [4]. To make things worse, some antibiotic-resistant pathogenic bacteria have evolved into multidrug-resistant (MDR) forms after the acquisition of resistance to multiple antibiotics. MDR, which continuously rises, may emerge by de novo mutation or, more frequently, by genetic recombination with a horizontally transmitted DNA sequence of an already resistant strain [5].

Animals, especially livestock, thanks to the non-therapeutic intake of antibiotics (to enhance growth and feed efficiency), play pivotal roles in generating and selecting ARGs worldwide. This leads to an increase in resistant strain populations that are first detected in animals and then excreted, along with antibiotic residues, whose load in wastes from animal husbandry industries exceeds that of anthropogenic habitats [6]. This is a very important issue because bacteria and ARGs often cross environments and species barriers, facilitating their spread and thereby amplifying the risks. Water, soil, and other habitats with highly varying ecological niches offer an unequaled, variable gene pool that considerably exceeds that of humans and domestic animals regarding the uptake of novel resistance factors [7,8].

Antibiotic resistance causes treatment failure and infections to relapse and drives increased morbidity and mortality with consequently amplified healthcare costs. Therefore, diminishing and preventing resistance development in a timely manner are acknowledged as pressing issues. Different strategies may be employed to find a solution for the problem of antibiotic resistance, such as the development of new bioactive-enhanced antibiotics (which were declined in the recent past), the development of vaccines to prevent infections (which are difficult to pipeline), the spread of awareness of these issues among the population (which is often misperceived), and primarily the therapeutic application of bacteriophages.

## 3. Bacteriophages

Bacteriophages or phages are viruses that infect bacteria and can transfer DNA horizontally. It is also possible to employ them to specifically kill bacteria and therefore combat antibiotic resistance without affecting animal cells. They represent an alternative to antibiotics to control bacterial-caused infectious diseases and try to fight the global threat posed by antibiotic resistance in bacterial pathogens.

The employment of bacteriophages as bactericidal agents dates back to 1919 after Felix d’Herelle and Frederick Twort independently discovered these viruses in 1917 and 1915, respectively [9,10,11]. Soon after, about a century ago, a period of enthusiastic development of commercial products containing bacteriophage doses was started, and they were applied to treat patients on a large scale. This period was followed by a time of declining enthusiasm for bacteriophage therapy in Western countries, which was concurrent with the global availability of antibiotics. Nevertheless, the development and application of phage therapy persisted in the Soviet Union and Eastern Europe [11].

With the advent of antibiotic resistance and, particularly, the global spread of MDR, phage therapy was re-considered worldwide in a more modern view and under the current regulatory standards to treat bacterial infections in humans and animals.

Bacteriophages, the most abundant organisms in the biosphere, can vary significantly in size (50–200 nm), morphology (polyhedral head without or with a collar and a contractile or noncontractile tail), and genome (from 3300 nt of RNA to 500 kbp DNA), with the majority being double-strand (ds) DNA tailed belonging to the former order *Caudovirales* [12]. Some bacteriophages are characterized by high specificity and restricted tropism for a particular strain within a certain bacterial species (narrow host range), while others bear a relatively wide host range and can infect various species within a bacterial genus or even members of different genera. The vast majority of bacteriophages, which carry a large dsDNA genome into an icosahedral capsid (or head) and a tail (former order *Caudovirales*), were formerly subdivided into three groups, namely *Myoviridae* (with a long rigid contractile tail), *Siphoviridae* (with a long flexible noncontractile tail), and *Podoviridae* (with a short noncontractile tail), which were recently removed as a classification, and they were all converted into the current class of *Caudiviricetes*. The remaining known bacteriophages have single-strand (ss) DNA genomes like the formerly known *Inoviridae* and *Plectoviridae*, which are currently classified as *Tubulavirales* or RNA genomes like dsRNA *Cystoviridae* and ssRNA *Leviviridae* (ICTV; https//ictv.global/taxonomy accessed 20 February 2024).

**Figure 1:**
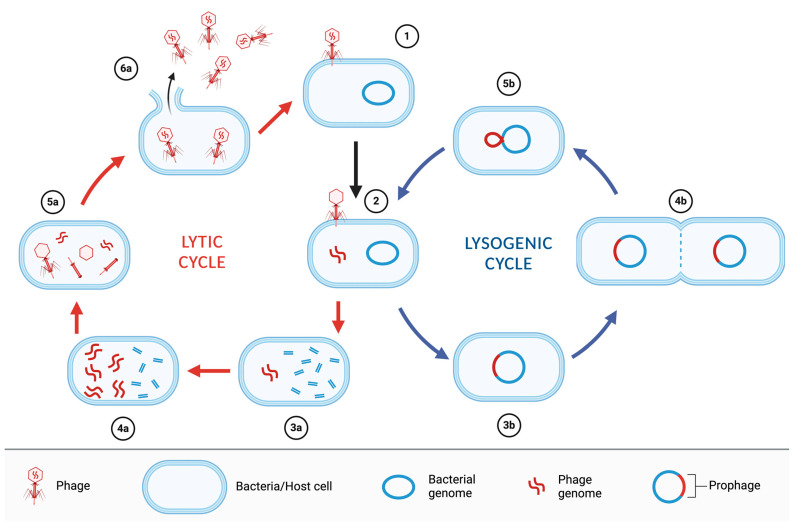
A schematic representation of bacteriophages’ replication strategy. After the attachment to the host bacteria (1) and the release of the phage DNA into the host cell (2), the lifecycle of a bacteriophage can follow two routes: the lytic cycle (left) or the lysogenic cycle (right). The lytic cycle includes the digestion of the host DNA by bacteriophage-encoded nucleases (3a), the synthesis and replication of the phage genomic DNA (4a), the expression of gene-encoded phage structural proteins and the assembly of new bacteriophage particles (5a), and cell lysis and the release of all phage virions (6a). The lysogenic cycle includes the integration of the phage DNA within the bacterial chromosome with the production of a prophage (3b), the reproduction of the lysogenic bacterium (4b), and the excision of the prophage from the bacterial chromosome (5b).

Bacteriophages are classified, according to their replication strategies, as lytic (or virulent) or lysogenic (or temperate) phages. Lytic phages, after the infection of the bacterial cell, hijack the cellular machinery, degrade the host cell DNA, and synthesize phage DNA and proteins to rapidly multiply and cause cell lysis and death in a short time, releasing hundreds of infectious viral particles that further infect other host cells (productive infection). Lysogenic phages, on the contrary, can integrate their genome into the bacterial chromosome after infection (or the genome can seldom stay as an episome) and remain latent (prophage), replicating along with the host cell chromosome and therefore be transferred to the progeny, even for a long time, until reactivation into a lytic cycle triggered by different stimuli (Figure 1) [11].

Since they are able to transfer genes from one bacterial strain to another, bacteriophages are defined as vectors, and this process, called transduction, can occur in a generalized or specialized manner. During generalized transduction, lytic phage capsids are filled with random fragments of bacterial genomic DNA instead of phage DNA [13]. On the contrary, in specialized transduction, lysogenic phages that have been integrated into the bacterial genome excise specific adjacent bacterial DNA sequences with their genome upon starting a lytic replication cycle, and the offspring of these phages transfer the same bacterial genes to their new hosts because lysogens have a specific integration site [14].

Obligate lytic bacteriophages have been considered more advisable for use as therapeutic agents since they quickly kill the target host cell. Conversely, temperate phages have been consensually avoided because of their intrinsic capacity to drive gene transfer among bacteria using specialized transduction, potentially helping the spread of antibiotic resistance or making bacteria pathogenic. Additionally, once integrated into the bacterial genome, they may exhibit superinfection immunity, making further phage administrations ineffective [14,15]. Nevertheless, in recent years, thanks to progress in sequence technology and system biology, temperate phages also started to be explored for gene therapy applications, generating encouraging results and envisaging the expansion of the armamentarium against the increasing antibiotic resistance threat. Potentially advantageous features of temperate bacteriophages are their abundance in nature (which makes them easy to find and isolate) and the possibility of engineering them to deliver genes that are lethal to bacteria or interfere with bacterial functions [14]. In this way, the risk of endotoxin release linked to the utilization of lytic phages could be reduced. It is even possible to remove genes tangled in the maintenance of lysogeny from the genome of temperate phages, transforming lysogenic phages into lytic ones [16]. Also, lytic phages can be engineered by removing the genes for endolysin production that are still inducing the death of the cell, but without the release of endotoxins that potentially cause fever and shock [17].

Well-annotated complete DNA sequencing of the genome of the phage intended to be used is always advisable in order to confirm the identity of the phage, predict the transduction efficiency, and exclude the presence of undesirable elements such as toxin genes or other features.

The advantages associated with phage therapy over antibiotics are as follows: (i) phages are able to increase their number when in the presence of bacterial targets and at the site of infection, where they are mostly needed (they have a self-replication property, which allows small doses and low costs), and then disappear once bacteria have been eliminated; (ii) phages minimally impact non-target bacteria or body cells (they have a narrow spectrum of action, which makes them harmless to the host organism and its commensal microflora); and (iii) phages can be very specific for their host strains. This third feature can be an advantage in minimizing the risk of secondary infections by not interfering with the endogenous commensal microflora. However, the narrow range of target bacteria can also be a disadvantage. To overcome this limitation, a mixture of multiple types of bacteriophages can be used. Cocktail formulations, mainly employing lytic phages, but also including temperate phages, may increase efficacy and minimize the rise of bacteriophage-resistant bacterial variants [18].

Lastly, bacteriophages are environmentally friendly compared to antibiotics.

The disadvantages of bacteriophage therapy are (i) the above-mentioned narrow spectrum of action; (ii) the potential interference with endemic bacteriophages; and (iii) the development of acquired resistance of bacteria to phages, which could hinder the success of the therapy. However, since bacteriophages co-evolve with their host, they can counter-adapt to the developed resistance strategies and, in the long run, phage-bacterium co-evolution may work in favor of bacteriophage therapy. To tackle the latter issue and develop more effective bacteriophage-based therapeutic approaches, it is crucial to expand the knowledge of phage resistance and to elucidate the mechanisms behind the emergence of resistance, which are still elusive.

## 4. Phage Therapy in Veterinary Medicine

Alongside the surge in publications focused on the many potential therapeutic uses of phages to treat human bacterial diseases, there has been a rise in the documented literature on the use of bacteriophages in veterinary medicine, especially for livestock (particularly cattle) and poultry. Comparatively fewer studies have been performed in companion animals, despite the increasing concern regarding the antimicrobial resistance of certain pathogens affecting pets and, potentially, their owners. Antibiotics provide an efficacious means for managing bacterial infections in farm animals, but using antibiotics either as growth promoters or as therapeutics for bacterial diseases in farming contributes significantly to the global health issue of antimicrobial resistance.

In human medicine, there are two different types of applications, namely compassionate use, which is personalized and restricted to individuals facing serious life risk due to the inefficacy of any other treatment, and randomized rigorous clinical trials [17].

### 4.1. Companion Animals

#### 4.1.1. Dogs

The increasing use of antimicrobials in pets, particularly dogs, including broad-spectrum molecules used in human medicine, promote antibacterial resistance, particularly among MDR bacteria, such as *Pseudomonas aeruginosa*, staphylococci, and *Enterobacteriaceae*. This issue should no longer be neglected in canine medical practice.

The most often isolated bacterium in canine otitis is *Pseudomonas aeruginosa*. Canine otitis with *P. aeruginosa* etiology is normally difficult to clear due to the resistance of *P. aeruginosa* to the most used antibiotics, making surgery for partial or full resection of the ear canal the only solution. Therefore, a more convenient and less invasive medical treatment, such as the use of bacteriophages, is foreseen as a necessity. The first publication reporting the use of phage therapy in dogs dates to 2006 and describes a successful case of virulent phage administration to a dog with chronic bilateral otitis externa caused by *Pseudomonas aeruginosa* resistant to antibiotic treatment. After the topical delivery of a suspension of phage that was previously tested in vitro for the complete lysis of the specific bacterial strain, there was full recovery without adverse effects or the further isolation of *Pseudomonas aeruginosa* up to nine months post-treatment [19]. Four years later, the members of the same research team released a second study, a real veterinary clinical trial, on the use of bacteriophage therapy for dogs with chronic otitis externa caused by *P. aeruginosa*. This study involved ten dogs receiving a cocktail of six bacteriophages tested for broad activity against different strains of *Pseudomonas aeruginosa*. The results, detectable 48 h after treatment, revealed a 30% reduction in clinical score coinciding with an increase in bacteriophage counts [20]. The long-term follow-up (18 months post-treatment) was very positive since, in the absence of side effects, six of the seven dogs that remained in the trial cleared out *P. aeruginosa* infection (three dogs were excluded because of other conditions and one still exhibited an ear infection despite surgery 1 year after phage therapy) [20]. Interestingly, the phage mixture was not tailored for each dog, suggesting that a standard product may be developed and commercialized for veterinary use.

In a recently published study, the virulent PEV2 phage characterized by low transduction potential and the ability to infect a wide range of *Pseudomonas aeruginosa* strains (candidate for human and canine phage therapy) was tested using a *Galleria mellonella* larvae model against a clinical isolate of *P. aeruginosa* from canine otitis; the results did not show a significant improvement in larval survival despite an increase in phage titer and lower bacterial proliferation [21], demonstrating that an in vivo model is critical for phage therapy and that the host (immune) system may play a crucial role in the final effect.

Successful attempts were made to isolate and characterize potential novel lytic bacteriophages that are effective against pathogenic multidrug-resistant strains of *P. aeruginosa* isolated from canine skin diseases [22]. Indeed, this opportunistic pathogen may be responsible for otitis externa in dogs, but also for chronic deep pyoderma, wound infection, and ocular infections such as ulcerative keratitis [22]. In vitro studies with numerous phages demonstrated that phages P5U5 and P2S2 displayed strong lytic activity against the wide range of *P. aeruginosa* strains isolated from canine ocular infections (100% and 80% lysis, respectively), and the combined preparation of both phages demonstrated a significant inhibition of bacterial growth at all MOIs tested [23].

Staphylococci, particularly *Staphylococcus pseudintermedius* and *S. intermedius*, are showing increasing antibacterial resistance, particularly methicillin resistance in dogs. Both species belong to the *S. intermedius* group. Methicillin-resistant strains of *S. pseudintermedius* (MRSP) and *S. intermedius* (MRSI) have been raising a major concern in canine medicine in the past decade [24]. The most prevalent skin illness in dogs, pyoderma, is associated in the majority of cases (up to 92%) with the opportunistic *S. pseudintermedius*, whose strains were revealed as methicillin-resistant in 59% of cases, and up to 98% of MRSP showed MDR to the antibiotics routinely used in veterinary practice [24]. *S. pseudintermedius* is a pathogen that is also present in other diseases, such as otitis externa, urinary and reproductive tract infections, and respiratory infections [24]. Therefore, it is extremely important to develop novel effective treatment options. Although no real veterinary clinical trials have been performed to test the efficacy of phage therapy against staphylococci in dogs, the data from in vitro explorative studies mainly focused on the isolation and characterization of bacteriophages targeting *S. pseudintermedius* and *S. intermedius* seem promising. To date, 19 phages that target *S. pseudintermedius* have been isolated and classified [25]; some have been characterized and showed lytic activity against MRSP [26], and some showed broad activity against methicillin-resistant species of different streptococci (*S. aureus, S. pseudintermedius*) and a lack of genes for toxins, virulence, and antibiotic resistance, which would make them candidates for bacteriophage therapy, except for lysogeny [27]. Lysogenic properties should be removed by mutagenesis and selection protocols. In an attempt to solve the problem of *S. pseudintermedius*, recently, two separate investigations examined the potential use of lytic *S. aureus* phages with host-range tropism for *S. pseudintermedius* isolates, but unfortunately, the selected *S. aureus* phages showed medium-low infectivity and low lytic activity for *S. pseudintermedius* [27,28]. Better results were obtained through the approach of isolating phage-encoded genes for endolysins, cloning, expressing, and purifying the enzymes for direct utilization to kill bacteria [28,29]. All of these studies are the basis for the future development of phage therapy against MRSP and demonstrate that more research is needed to understand and treat these pathogenic antibiotic-resistant staphylococci in dogs.

Another potential use of phage therapy is for urinary infections targeting the upper (kidney and adjacent ureter) or the lower (bladder and adjacent urethra) tract, which are often diagnosed in dogs and commonly caused by *Escherichia coli* or other bacteria from the intestinal microflora [30]. The urogenital apparatus of dogs harbors a large community of antimicrobial-resistant isolates of *E. coli*, which have a tight exchange of resistance genes with their phages [31]. In the context of scanty information and a limited choice of molecules for treatment, MDR *E. coli* strains represent a serious health issue not only for dogs but also for humans, with dogs being carriers of the pathogen [32]. A study aiming to identify new bacteriophages from naturally occurring *E. coli* infections in dogs and cats showed that more than 90% of the ten bacteriophages isolated were able to lyse about 50% of the target *E. coli* obtained from canine and feline feces when singularly tested, and more than 90% were able to lyse the target when mined in a cocktail [33]. This result is encouraging because it shows that the majority of uropathogenic strains of *E. coli* in dogs and cats are sensitive to these phages; therefore, they are potentially useful for phage therapy.

Antibiotic resistance is a rather widespread problem today that also affects dogs; to solve this problem, new methods must be developed. Although there has only been one clinical trial of phage therapy in dogs (targeting *P. aeruginosa*) to date [20], encouraging data obtained in an in vitro study show the possibility of using it for various infections such as the ones caused by *S. pseudintermedius* and *E. coli*.

#### 4.1.2. Cats

No real veterinary clinical trials have been reported to date on the possible application of bacteriophage therapy in cats. Only an in vitro study is available on cats, in conjunction with dogs, on the uropathogenic strains of *Escherichia coli*, as reported above [33]. Cats, like dogs, are prone to urinary tract infections commonly caused by antibiotic-resistant *E. coli*, which are difficult to treat and often relapse. Therefore, alternative approaches to antibiotics are urgently needed to target this and other diseases in cats with bacterial etiology. In this context, phage therapy may be beneficial.

#### 4.1.3. Horses

In horse clinics, the incidence of nosocomial infections and surgical site infections caused by multi-resistant pathogens is dramatically increasing. Hence, actions are required, and within them, phage therapy may be strategic to defeat antibiotic-resistant bacteria, such as MDR *Staphylococcus aureus* and *Klebsiella pneumoniae*.

Only one real veterinary clinical trial has been performed in horses, particularly against *Staphylococcus aureus* present in equine superficial bacterial pyoderma [34].

Equine pyoderma with staphylococcal etiology is commonly detected as a major clinical condition in horses treated with antibiotics. In particular, *Staphylococcus aureus* is the most frequently isolated pathogen among the staphylococci found in horses’ skin lesions, and the one that has the most resistance to antibiotics [35]. Treating superficial bacterial pyoderma in horses might be difficult due to the need for prolonged treatment periods and high doses of systemic antibiotics to adequately reach the skin, with adverse effects often implicated. A recently published pioneering study demonstrated that a cocktail formulation of two bacteriophages specifically targeting *S. aureus* that was topically administered to a cohort of 20 horses with *S. aureus* superficial pyoderma resulted in the target bacteria being killed, but allowed the overgrowth of commensal or other cocci, resulting in a lack of improvement in the clinical score and no reduction in the total number of bacteria or in the inflammation cell (neutrophils) count at the infection sites throughout the 28-day study period [34]. Probably, since *S. aureus* is not the only harmful bacterium in horse pyoderma, it would be advantageous to make bacteriophage cocktails with a wider variety of phages. Overall, this pilot investigation demonstrated that a specifically targeted organism may be killed by a topical bacteriophage, but other important aspects are also management, environment, and the search for all possible causes of pyoderma.

Another severe disease reported in horses is pneumonia caused by MDR isolates of *Klebsiella pneumoniae*, a putative zoonotic pathogen [36]. It was recently demonstrated that multiple clones of *K. pneumoniae* carrying different antibiotic resistance genes can be co-present in a single episode of infection, with each clone being sensitive to the lytic activity of different bacteriophages [37]. This study, which emphasizes the use of bacteriophage typing as part of routine testing, is a basis for the identification and characterization of new phages with lytic activity that might be considered for future application against MDR *K. pneumoniae*.

### 4.2. Livestock

#### 4.2.1. Ruminants

Phage therapy also has the potential to be an effective treatment for bacterial infections in ruminants, particularly cattle and sheep. Ruminants are vulnerable to a variety of bacterial diseases, which can result in severe economic losses for farmers and a negative impact on animal wellbeing. Moreover, ruminants may also be reservoirs for zoonotic pathogens. Therefore, the research around bacteriophages led to their application to some ruminant bacterial infections with *Staphylococcus aureus*, *Escherichia coli*, and *Listeria monocytogenes* as etiologic agents.

Bovine mastitis is the most crucial threat to dairy cattle farmers for both economic (reduced quality and quantity of milk production) and health (poor cow health and potential fatality) reasons [38]. Among the most common bacteria isolated from contagious clinical and subclinical mastitis is *Staphylococcus aureus*, against which the current antibiotic therapy option is mostly inefficacious because of methicillin-resistant *Staphylococcus aureus* (MRSA) strains [38]. The first clinical trial attempting to treat antibiotic-resistant *S. aureus* with phage therapy dates back to 2006, where the virulent phage K was employed [39]. Major limitations were highlighted, such as the inactivation or degradation of the phage by the immune system and some components of the milk, which caused the treatment to be ineffective. Clinical trials not in cows, but in both *Galleria mellonella* larvae and mice models were more successful when three of four in vitro-characterized lytic bacteriophages anti-*S. aureus* were tested, resulting in a significant improvement in both models [40]. Different virulent phages (among which MSA6) were then newly isolated from cows with mastitis and showed in vitro features that are potentially attractive for phage therapy, such as high lytic activity against numerous pathogenic and antibiotic-resistant strains of *Staphylococcus aureus* and thermostability [41,42]. Nevertheless, they were not employed in clinical trials. An even more alternative approach was an in vitro-assayed mix of three lytic bacteriophages in conjunction with the lactic acid bacterium *Lactobacillus plantarum* to target *S. aureus* [43]. Such a strategy, which foresees the combination of the antibacterial activity of the probiotic *L. plantarum* with the phage cocktail, showed the promising result of higher in vitro antimicrobial activity 24 h post-application to challenge *S. aureus* isolates from bovine mastitis cases [43]. Obviously, additional investigations are necessary, as well as a longer observation time and an in vivo trial, but these results seem encouraging anyway.

As an alternative to the use of bacteriophages, due to their excellent specificity, restricted range but quick antibacterial activity, minimal likelihood of target organism resistance, and non-transmissibility of virulence factors, bacteriophage-derived lysins are being investigated in vitro in natural or engineered chimeric forms for their ability to target *S. aureus* [44,45].

Another bacterium causing bovine mastitis is *Escherichia coli*, against which three lytic phages (SYGD1, DYGE1, and SYGMH1) were isolated from a sewage dairy farm and used to prepare a cocktail, which was tested in heifers that were pre-inoculated with antibiotic-resistant pathogenic *E. coli*. The results evidenced significantly lower numbers of bacteria, somatic cells, and inflammatory molecules and milder symptoms of mastitis. These phages, although unable to achieve the complete clearance of bacteria, seem promising because of their ability to control bacteria, and they also remained stable at wide ranges of pH and temperature [46].

Another important bacterium infecting calves, especially in the early weeks of life, is Shiga-toxin-producing *Escherichia coli*, which causes neonatal diarrhea. Difficulties in defining the right antibiotic and duration of treatment, along with increasing antibiotic resistance, led to the development of an alternative strategy based on treatment with bacteriophages together with the probiotic bacterium *Lactobacillus* spp. to target Shga-toxin-producing *E. coli* strains. Suppositories containing *Lactobacillus* spp. and a cocktail of three *E. coli* lytic bacteriophages (φ26, φ27, and φ29) previously selected in vitro were tested in vivo in calves that were a few days old with the aim of evaluating the effect against neonatal diarrhea. The results demonstrated both therapeutic and prophylactic effects of probiotic-phage suppositories since diarrhea was eliminated within 48 h and for 11 days, with no impact on the endogenous microflora [47]. This promising procedure may be employed for other animal species and humans.

Not only neonates with diarrhea, but also healthy calves, as well as sheep, can carry the Shiga-toxin-producing *Escherichia coli* O157:H7 despite not having pathologies, and they can spread it through fecal excretion. The problem is that *E. coli* O157:H7 is a significant zoonotic foodborne pathogen for humans, where it possibly causes severe bloody diarrhea, hemorrhagic colitis, and hemolytic uremic syndrome. The main reservoir is represented by these ruminants [48]. Back in the 1980s, rigorous studies on phage therapy to treat infections with pathogenic *E. coli* etiology (and particularly neonatal diarrhea due to enterotoxigenic *E. coli* strains and septicemia) started in calves and demonstrated that the use of bacteriophages in vivo, through oral administration, leads to a significant pathogen reduction without adverse effects on the treated animals [49,50,51]. These studies, which are definitely noteworthy, still left some basic aspects unclear, such as pharmacokinetics and preparations (crude and not purified). More recently, a study that was performed both ex vivo and in vivo with a cocktail of two phages targeting *E. coli* O157:H7 orally administered to cattle showed a certain decline in fecal bacterial shedding in the 24–48 h after treatment, but the results were not statistically significant [52]. Similarly, a trend toward treatment effect was observed in steers that were orally administered a cocktail of four lytic bacteriophages targeting *E. coli* O157:H7 [53]. When the same research group compared oral versus rectal administration of the cocktail, including the same four lytic bacteriophages targeting *E. coli* O157:H7 in feedlot steers, it was found that the oral route was (unexpectedly) more efficacious [54]. A previous investigation had already highlighted limited success of the recto-anal application of a mix of the two *E. coli* O157:H7-specific lytic KH1 and SH1 phages in steers (and continuously provided in drinking water), as it only reduced and did not cleared the challenge bacteria in most animals [55]. The same phage cocktail was orally tested in sheep, and a reduction in *E. coli* O157:H7 shedding was also not observed in this species [55].

Few other studies were performed on sheep. One study, which employed both an artificial rumen system (Rusitec) and experimental sheep, demonstrated that the administration of the single specific *E. coli* O157:H7 phage DC22 completely cleared out the bacterium from the bioreactor in 4 h, but no effect was obtained in the lambs after oral administration [56]. A slightly more successful result was achieved by orally administering a different single phage (the T4-like bacteriophage CEV1) to ewes as the treated animals showed reduced *E. coli* O157:H7 cells in the caecum and rectum, and this result was seen at lower extent in rumen two days post-treatment [57]. Better results were obtained a few years later by the same research group when a cocktail containing a mix of two bacteriophages, CEV1 and CEV1, respectively, T4- and T5-like, was orally inoculated to ewes, and this led to a significant diminishment in *E. coli* O157:H7 in the entire low intestinal tract without showing side effects [58].

Overall, these clinical trials demonstrate that single phages are low or not effective in killing all of the target bacteria, while cocktails of multiple phages are more effective; nevertheless, bacteriophage cocktails do not always show complete efficacy. In addition, the oral route of administration represents an easy farmer-applicable type of treatment but may require the protection of the phages (encapsulation) from the acidic pH and the proteolytic enzymes of the stomach, which may affect their activity. Indeed, two different systems of polymeric encapsulation applied singularly and in a mix of four anti-*E. coli* O157:H7 bacteriophages for oral or in-feed delivery were developed and tested in steers but failed to limit bacterial shedding [59]. Additionally, other aspects should be taken into consideration, such as other phages that are naturally present, which can be activated to replicate when the massive arrival of challenge bacteria occurs. Host selectivity is an additional parameter that is crucial for the choice of phages to combine in the cocktail. Finally, a detailed characterization of the phages on one side and a better knowledge of the host gut ecosystem on the other side should be considered a crucial requirement for developing an effective phage therapy technique against pathogenic strains of *E. coli*.

Similarly to *E. coli*, *Listeria monocytogenes* is also a food-borne zoonotic pathogen found in dairy cattle farms, which represent a reservoir. Moreover, similarly to *E.a coli*, *L. monocytogenes* is also characterized by resistance to multiple antibiotics. Still, in the absence of clinical trials, to pose the basis for developing bacteriophage therapy against multidrug-resistant strains of *L. monocytogenes*, six (LMP1-LMP6) new phages were recently isolated and characterized for the host range and stability at wide ranges of pH and temperature. This led to the selection of one phage (LMP3) being more suitable for killing multidrug-resistant *L. monocytogenes* [60]. The in vitro conjugation of this phage with silver nanoparticles further demonstrated enhanced anti-*Listeria monocytogenes* activity and more stability [60]. New technologies, such as nanotechnology, may therefore be used to develop carriers of bacteriophages that are able to inexpensively improve stability and help to combat multidrug-resistant *L. monocytogenes*.

#### 4.2.2. Pigs

Pigs are susceptible to a variety of bacterial infections, which can have a negative influence on their health, growth, and production.

The first investigations of bacteriophage therapy in pigs date back to the 1980s when the efficacy of two lytic phages applied singularly or mixed in a cocktail was tested to treat the economically impacting neonatal diarrhea caused by enterotoxigenic strains of *Escherichia coli* (ETEC) in piglets [49]. The results were encouraging since the neonatal diarrhea was mild and none of the pigs died contrary to the controls; however, unfortunately, phage-resistant bacteria developed. In the subsequent years, the interest in phage therapy for this application diminished concurrently with the development of fimbriae-based vaccines [61] and finally resumed a few decades later, when the antibiotic resistance of ETEC rose. Nine different bacteriophages were isolated and characterized, and in vitro assays demonstrated the suitability of six of them (GJ1-GJ6) for phage therapy against different types of ETEC since they were active and lacked genes for toxins and lysogeny [62]. The six selected phages were further tested in vivo in piglets, and three of them showed that after oral administration carried out singularly or in a mix, the conditions significantly improved without causing alterations to the normal microflora [63].

In order to avoid the potential disruption of the phage due to the acidic and proteolytic stomach environment, phage A221, previously selected in vitro, was microencapsulated with sodium alginate and tested in vivo in piglets. After oral administration to weaned piglets that were pre-challenged with *E. coli*, the results showed significantly improved conditions and reduced bacterial load in the treated animals versus the controls. This trial did not include a group with a non-incapsulated phage because it was previously demonstrated in vitro that it is inactivated under gastric conditions; however, a group of animals was treated with antibiotics to compare the effect of phage versus antibiotic therapy. Despite the impossibility of gaining insights into the effect of microencapsulation on the results obtained, it could be concluded that the treatment with the bacteriophage reached the same efficacy as antibiotics without impairing the gut microflora and with the absence of toxicity [64].

Pigs are food animals and therefore reservoirs for zoonotic pathogens, such as *Salmonella* spp., *Staphylococcus aureus*, and *Streptococcus suis*.

The foodborne pathogenic bacterium Salmonella is responsible for salmonellosis in humans and pigs, which is associated with morbidity and mortality. In a One-Health view, to try to limit the entry of Salmonella into the food chain, bacteriophages isolated from the feces of commercial finishing pigs and characterized for being anti-*Salmonella enterica* serovar Typhimurium were orally administered as a cocktail to weaned pigs. The results showed a reduced specific bacterial load in the intestine but also highlighted the need for more information to optimize the preparations, doses, and administration schedule [65]. Since the *Salmonella enterica* serotype Choleraesuis is the most frequent cause of salmonellosis in pigs, a recent in vitro investigation was conducted with the aims of isolating lytic bacteriophages specifically acting against *S.* Choleraesuis from canals and slaughterhouse drainage water and characterizing and testing them in simulated intestinal fluid to verify the efficacy of applying them singularly or combined in a cocktail [66]. The results also showed in this case that the bacteriophage cocktails (including more than two phages) were more effective in infecting and killing the target bacterium than when a single phage was administered. Indeed, cocktails remedy the problem of restricted host range and yield a lower resistance development rate. Moreover, the study highlighted the importance of resistance to temperature and pH for bacteriophage candidates for orally administered therapy. In order to address the gaps in the utilization of bacteriophage therapy on a commercial large scale, such as pig farms, a research group investigated the possibility of phages to survive in the milling process and tested dry feed containing a cocktail of the two lytic bacteriophages SPFM10 and SPFM14, which was prophylactically administered (before the challenge with *S.* Typhimurium) [67]. The results showed a significant reduction in target bacteria colonization in all of the gastrointestinal tracts of the pigs that were prophylactically fed phage-feed diet, with no impact on the endogenous microflora.

Pigs, similar to or even more so than ruminants, are reservoirs of *Staphylococcus aureus* and particularly MRSA, posing a serious One-Health concern worldwide. To date, only one study has been published on the use of a cocktail of bacteriophages (K*710 and P68) to fight MRSA in pigs [68]. The study included in vitro, ex vivo, and in vivo models to determine the efficacy of phage therapy against the MRSA nasal colonization of pigs. The results showed that the target MRSA was killed in the in vitro model, while ex vivo (on swine nasal mucosa explants) and in vivo, the phage cocktail did not result in any reduction in the MRSA cell count [68]. This study points out that extensive research is required to obtain a complete grasp of phage–bacterial interactions in vitro before beginning in vivo studies. Moreover, it demonstrates that ex vivo experiments may be very useful after in vitro studies and before in vivo ones to obtain valuable data.

*Streptococcus suis* is a relevant swine pathogen that is transmissible to humans. This emerging zoonotic agent may cause meningitis, arthritis, endocarditis, sepsis, pneumonia, and sudden death in both pigs and humans. The infection causes severe economic losses in the pig industry and raises major concerns from a One-Health perspective. To tackle the continuous emergence of strains that are resistant to antibiotics and to limit the spread of the pathogen in the absence of bacteriophage trials, phage-derived lysins have been considered. The lysin produced by the phage SMP (lytic for *S. suis* serotype 2) was tested in combination with an antibiotic and bacteriophage on a biofilm created by *S. suis*. The results showed almost a complete (80%) disruption of the biofilm; therefore, it was much more efficient than the bacteriophage or the antibiotic alone, which dispersed less than 20% of the biofilm [69]. More recently, a research group isolated two new lysins from lysogenic phages (phi7917 and phi5218) targeting *S. suis*, which are tolerant to wide ranges of pH and temperature. They displayed efficient lytic activity both in in vitro and in vivo (mouse) models against multiple serotypes of *S. suis* [70,71]. The lysin from phage phi5218, which was the most effective in vitro and in the mouse model, was then tested in vivo in piglets, demonstrating the therapeutic potential in controlling different serotypes of *S. suis* [72].

Still, additional work should be conducted to improve the application of phage therapy in pigs. Doses, the preparation and formulations suitable for field applications, as well as the evaluation of the results under field conditions are the main issues to be addressed along with increased knowledge of the bacteriophage virulence characteristics and phage–host interactions.

#### 4.2.3. Poultry

**Figure 2:**
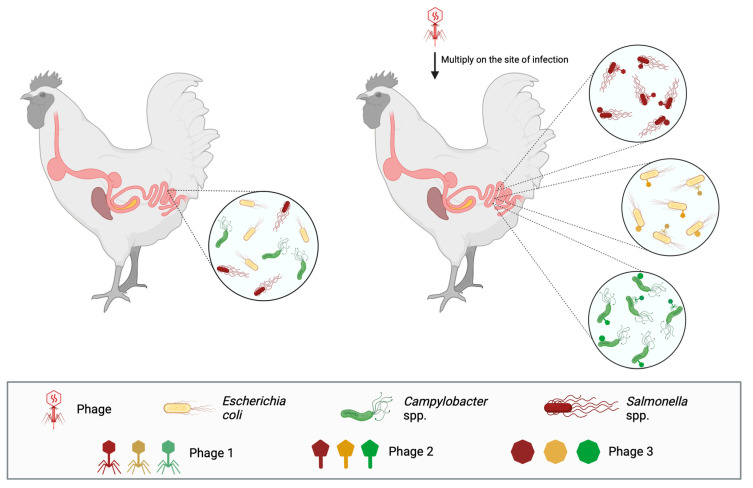
Bacteriophage therapy has been developed to treat gastrointestinal diseases in poultry, caused by *Salmonella* spp., *Campylobacter* spp., and *E. coli*. In the left panel are the bacteria, and in the right panel are the cocktails of bacteriophages used to target the specific bacterium and treat the corresponding infectious disease.

After the appearance of antibiotic-resistant bacteria and antibiotic residues in poultry-derived food, bacteriophage therapy has been applied in poultry against pathogens that, besides being known to cause diseases and economic burden, are also zoonotic and find poultry as a natural reservoir, such as *Salmonella* spp., *Campylobacter jejuni*, and *Escherichia coli* (Figure 2).

*Salmonella enterica* serovar Enteritidis and *Salmonella enterica* serovar Typhimurium represent major heath concerns because they are zoonotic agents and, respectively, the second and the first most commonly isolated serovars from human salmonellosis. These two serovars took over, especially in developed countries, after the eradication of *Salmonella enterica* serovar Gallinarum, which can cause severe acute systemic illness in young chicks with a high mortality rate.

A series of experiments was conducted that singularly tested three different lytic phages, with each one being used for a different serovar of *Salmonella enterica* that was therapeutically administered by oral gavage in broilers previously challenged with the specific bacterium, and they demonstrated efficacy in significantly reducing the number of target bacteria in the caecum for two of the three serovars (Enteritidis and Typhimurium). Still, they raised bacteriophage resistance proportionally to the titer of phage used [73]. An attempt to target *S. enterica* serovar Enteritidis was performed with cocktails of *S.* Enteritidis-specific lytic bacteriophages (isolated from poultry and human sewage sludge) in vitro and in vivo. The results from both the in vitro model and in the experimentally infected broiler chicks inoculated by oral gavage demonstrated transient effectiveness, with a significant reduction in the target bacterial count only at 24 h, but not at 48 h post-treatment. In addition to the short-lived effect, this study showed that the administration of bacteriophage cocktails alone or in combination with probiotics did not impact the results [74].

The administration of a cocktail with multiple phages may be simultaneously effective against the two most common serovars of *Salmonella*, Enteritidis and Typhimurium, as demonstrated in a trial involving a cocktail of three broad-range bacteriophages that were orally administered to chicks. This study also demonstrated that repeated administrations of the phage cocktail, particularly the administration of the cocktail before the challenge, resulted in a significant decrease in the pathogenic bacteria count in the caecum and more efficacy than the trials where phage therapy was administered a few days after challenge bacteria infection and colonization [75].

Another study confirmed that preventive therapy, even with a single orally administered phage, can cause a reduction in fecal Salmonella. Moreover, in the same study, the delivery of non-encapsulated phages versus encapsulated ones (in two different types of polymers) showed that encapsulation did not influence the survival of the phage in vivo through the proventriculus and gizzard of the young chick despite the observation that, in vitro, the non-encapsulated phage did not survive the simulated gastrointestinal conditions [76]. This indicates that the in vitro results should be cautiously considered because the in vivo phage–bacterium interaction may be quite different from the in vitro interaction. The benefit of encapsulating phages is controversial and probably depends on the type of phage biology and the encapsulation method. Indeed, contrarily to the previously reported study, a recent investigation on the encapsulation of a Salmonella-specific lytic phage with xanthan gum/sodium alginate/calcium chloride/chitooligosaccharides proved to be effective in reducing *Salmonella* Enteritidis both in vivo and in vitro compared to the non-capsulated one [77].

Since the administration of bacteriophages before the challenge with the target bacteria has shown that preventive therapy is more efficacious than post-infection therapy, recent investigations adopted this scheme. A study tested the efficacy of three different doses of a cocktail made of the two *Salmonella* Typhimurium virulent phages SPFM10 and SPFM14 that was administered in feed to a large scale of broiler chicks that were challenged 4 days later with *Salmonella* Typhimurium. The results showed a reduction in Salmonella colonization in the chicks that were pre-treated with the bacteriophage at all doses along with better growth performances and increased body weight gain [78]. Thus, this could be a viable option for therapy administration in commercial farms.

Few long-term in vivo trials, despite the absence of complete clearance, gave statistically significant results of target bacteria reduction. One study that used a single lytic phage through oral treatment showed a diminished *S. enterica* serovar Enteritidis load in the cloaca of young chickens up to 14 days after treatment [79]. Another long-term in vivo study on broiler chicks employed a cocktail of three different bacteriophages isolated from the feces of free-range chickens and showed that, after oral administration, a 3.5-fold reduction in the *S. enterica* count could be measured in the feces, which lasted for 25 days [80]. A research group recently demonstrated that a mix of two phages, a virulent one and a temperate one targeting *S.* Typhimurium, that was orally administered to Salmonella-free chicks after experimental infection with a multidrug-resistant *S.* Typhimurium strain resulted in a great reduction in the bacterial load in 10 days later but could not prevent the symptoms in some organs and could not restore the normal intestinal microbiota composition [81].

Finally, in a commercial farm, multiple long-term and large-scale trials recently reported a positive effect of a cocktail of three bacteriophages targeting *Salmonella* that was administered for 16 consecutive days by oral gavage to a large number of commercial broilers that were challenged 17 days later by oral gavage with a suspension of five different Salmonella serovars. This “preventive” therapy allowed for the detection of no prevalence of *Salmonella* in the cloaca at some time points, while the administration of the phage cocktail after the challenge only caused a reduction in the bacterial load. The bacteriophage therapy also induced an increased body weight as an indirect effect. Overall, the results were durable and highly effective, suggesting the practicability of this approach in commercial farms [82].

A few studies focused on *Salmonella enterica* serovar Gallinarum were conducted. A mix of three bacteriophages (isolated from sewage water) with broad serovar host ranges that was orally inoculated as a feed additive a week before the challenge with *S.* Gallinarum proved to be effective in reducing or blocking the horizontal transmission of the pathogen in layer chickens and therefore contained fowl typhoid [83]. Recent research addressed the vertical transmission issue. It showed that the oral administration of the single lytic phage CKT1 isolated from farm sewage to *S.* Gallinarum biovar Pullorum experimentally infected broilers and significantly reduced the bacterial load in the reproductive tract, in the eggs, and on the eggshell, and also diminished *Salmonella*-specific immunoglobulins G in the serum of the challenged animals [84]. The way in which phages can cross the intestinal barrier and reach the reproductive apparatus is still largely unknown; regardless, this is the first study that proved the potentiality of phage therapy in controlling the vertical transmission of bacterial infection in poultry.

Finally, research on bacteriophages that are efficacious as therapeutics against MDR Salmonella may also benefit from research that is conducted with the aim of destroying biofilms composed of Salmonella. Phages that display lytic activity against Salmonella-derived biofilms, such as the recently identified and successfully tested UPF_BP1 and UPF_BP2 specific for *S.* Gallinarum, or BP1369 and BP1370, respectively, targeting *S.* Typhimurium and *S.* Enteritidis in biofilms may be tested for their potential usefulness and to control the pathogen in the birds [85,86].

Poultry often host *Campylobacter* as a commensal, which normally does not have pathological effects on them, but they are reservoirs for this enteric pathogen in humans, which has lately been complicated by its demonstrated resistance to different antibiotics. The dissemination of *Campylobacter* occurs mostly at the slaughterhouse; therefore, it is necessary to reduce the presence of *Campylobacter* in the avian species in order to limit its entry into the food chain.

*Campylobacter jejuni* and *Campylobacter coli* are the two most common species of *Campylobacter* against which bacteriophage therapy approaches were developed. A trial was performed by administering a single dose of the virulent phage CP220 to broiler chicken that were previously infected with *C. jejuni* or *C. coli*. The results showed a significantly lower target bacteria count in the intestines of both species, but *C. coli* required a higher dose of phage [87]. A cocktail of three phages with a wide lytic spectrum against *C. jejuni* and *C. coli* was tested in vivo in broiler chicks that were previously infected with *C. jejuni* or *C. coli.* The results demonstrated a reduction in the target bacterial count persisting for the duration of the trial (7 days) [88]. Moreover, this study investigated, for the first time, the administration route, and particularly compared oral gavage with administration in feed, showing that the administration route also has an impact on the output; specifically, the administration in feed causes an earlier reduction in the target bacterium [88]. This method of phage therapy application, incorporated into dry feed, is especially advantageous and practical for the poultry industry. In a different study, preventive versus treatment administration by oral gavage of a single lytic phage to broiler chickens for 10 consecutive days resulted in a similar decrease (by several orders of magnitude) in the bacterial load in the caecum in comparison with the untreated group (except that the prevention delayed the challenge bacteria colonization) [89]. When a cocktail of two different lytic phages was therapeutically administered to adult chickens for four consecutive days after a challenge with *C. jejuni*, the treated birds displayed a lower caecal load of bacterium than the controls [89]. In the first investigation conducted in the field, four bacteriophages were given in a cocktail in four different commercial broiler flocks through drinking water and allowed to contract *Campylobacter* naturally [90]. The first commercial trial showed a statistically significant decrease in fecal bacteria that was detectable in broilers treated (through the drinking line) with a lytic bacteriophage cocktail in comparison to the controls. In this trial, the effect was detectable starting from 1 day post-treatment to slaughter; no significant reduction was observed in the other trials [90]. A decade later, the effectiveness of the administration of a cocktail of two lytic phages through drinking water in a field-condition trial in broiler chicks that were pre-infected with *C. jejuni* was confirmed [91]. These studies raised issues about standardization and reproducibility, which may have been resolved by administering a cocktail of broad-spectrum bacteriophages and for a longer time. Moreover, the application of phage therapy to hens or production broilers inevitably entails the release of phage-infected *Campylobacter* in the environment and is a risk that should be assessed. Another consequence of bacteriophage therapy, namely the recovery of some resistant phenotypes, which do not impede the reduction in *Campylobacter*, has been confirmed by another research group after experimentally infecting broiler chicken with *C. jejuni* and then treating them with a cocktail of two lytic phages (by oral gavage) [92]. Therefore, the choice of the appropriate phage and the optimal dose are crucial aspects to consider.

A meta-analysis recently conducted on the efficacy of phage therapy in poultry showed that bacteriophage delivery can significantly diminish the target bacterial load in poultry and that the efficacy, which varies according to the administration route, is generally greater in short versus long times and in older versus younger chickens; it can be effective when administered either as a prophylactic or therapeutic, independently from the number of doses [93].

In order to reduce the bacterial count at slaughter and to also diminish the development of phage-resistant bacterial strains, a research group tested the efficacy of a two-step single-phage application of field-isolated lytic phages specific for antibiotic-resistant *C. jejuni* to previously challenged adult broiler chickens two days before slaughter. The results demonstrated a statistically significant decline in the bacterial count [94]. This innovative approach, which seems encouraging to achieve reductions in bacterial spread and antibiotic resistance at the same time, highlighted that the timing of bacteriophage administration is crucial for the success of the therapy and the development of resistant mutants as well.

Colibacillosis is an economic challenge for poultry production, often beginning as a respiratory infection and then becoming airsacculitis, septicemia, enteritis, osteomyelitis, and peritonitis. Antibiotics have been extensively used against avian pathogenic *Escherichia coli* and have contributed to select antibiotic-resistant strains. In addition, pathogenic *Escherichia coli* antibiotic-resistant bacteria may pass directly or indirectly to other animals and humans, posing significant risks from a One-Health perspective.

An early study found that the intramuscular inoculation of the lytic phage R can prevent and treat septicemia and meningitis in both chicks that are newly hatched and a few weeks old that were experimentally infected with the K1-positive bacteremic strain of *E. coli*. In addition, the bacteriophage was able to prevent death from meningitis caused by the intra-cranial inoculation of the same bacterium. Phage administration given a few days before the challenge worked as a prophylaxis as well as if given therapeutically at the onset of the clinical signs. This study confirmed that if bacteria are present in both the blood and brain, the bacteriophage can cross the blood–brain barrier [51].

Different ground-breaking experimental trials were performed to cure chicken airsacculitis with bacteriophage therapy. One set of trials were conducted to compare the inoculation of a single bacteriophage (SPR02) into the air sacs of chicks mixed with the challenge *E. coli* versus the bacteriophage administration in drinking water. The results demonstrated that the phage administration in the drinking water had no effect or protection when the phage was inoculated into the air sacs and mixed with the challenge bacteria [95]. Although this experiment was artificial, it demonstrated, for the first time, the premise to combat airsacculitis. The same research group then tested the efficacy of a mix of the previously tested phage (SPR02) with a second lytic phage (DAF6) administered by aerosol spray. The results showed a certain degree of protection against the challenge pathogenic *E. coli* as the mortality significantly decreased; however, no complete protection was achieved [96]. When aerosol spray was compared with intramuscular injection, the first route demonstrated much less effectiveness than the second one [97]. Finally, when the cocktail of the two above-indicated bacteriophages was administered in combination with an antibiotic (Enrofloxacin), the phage therapy demonstrated to be a bit less effective than the antibiotic, but the synergy of the two treatments resulted in a significant improvement [98]. Clearly, the effectiveness of the phage treatment depends on the route of administration and the titer, being more effective when an adequate amount of bacteriophage reaches the critical site of the bacterial infection. This was confirmed by other researchers, who treated severe respiratory *E. coli* infections with a single tracheal inoculation of a mixture of three lytic phages (phi F78E, phi F258E, and phi F61E) immediately after the challenge [99].

A newly isolated and characterized lytic phage that is active against multiple strains of pathogenic *E. coli* was tested in vivo to determine the therapeutic and prophylactic effect against a multidrug-resistant avian pathogenic *E. coli* strain. The results of the therapeutically applied (orally fed) phage showed a 20% reduction in mortality compared to the controls, while the prophylactic administration (the phage was orally fed before bacterial challenge) resulted in a 30% reduction in mortality compared to the control group. Moreover, the treated animals displayed a significantly higher body weight gain, and at the post-mortem analysis, the phage was present in the air sacs and the lungs, proving that oral administration is adequate for the phage to reach the target organs [100]. These preliminary results are encouraging but highlight the need to address quite a few clinical practice issues.

Another study reported the isolation and characterization of phage CE1 and the efficacy of the phage intramuscular injection 2 h after challenge injection with a high pathogenic avian *E. coli* in reducing the target bacterial load in 1-week-old broilers. The efficacy of the phage therapy was a bit better than the one obtained with antibiotics [101].

Few experimental trials employing bacteriophages were performed with the aim of treating enteropathogenic strains of *E. coli*. One study employed the bacteriophage Esc-A to orally treat newly hatched chicks and compared the results with the common antibiotic therapy. The results of the phage therapy were far better compared to the antibiotic treatment since diarrhea disappeared starting from the second week, the death rate was the lowest, and the animals increased their body weights without any impact on the intestinal microflora [102].

A new and alternative strategy that was recently developed to control avian pathogenic *E. coli* is based on the administration of bacteriophages in ovo. Two different trials were carried out by the same research group, and both gave promising results. First, they isolated, characterized, and selected eight bacteriophages belonging to eight different genera and mixed them in a cocktail. The cocktail was inoculated in embryonated eggs on day 12, 2 h after the inoculation of the challenge pathogenic *E. coli*. The embryonated eggs, candled for the following 6 days, showed 90% protection from death compared to the control eggs, which presented 100% mortality [103]. In the second study, a bacteriophage cocktail inoculated in ovo was evaluated for its efficacy in protecting chicks from colibacillosis caused by avian pathogenic *E. coli*. Four of the eight previously used phages were mixed in a cocktail, which was inoculated into embryonated eggs (amniotic or allantoic fluid) at day 17, and 1 day after hatching, the chicks were challenged with avian pathogenic *E. coli* and. Two of the four phages were found in the caecum of the chicks on day 7 post-hatch, and the chicks treated with the bacteriophages did not develop colibacillosis lesions. The pathogenic bacteria retrieved from the intestines were significantly lower than those from the controls [104]. These investigations proved that the in ovo inoculation of phages (better in amniotic fluid) can cause protection against colibacillosis despite not achieving clearance of the target bacteria. A drawback of this approach is that the majority of the surviving bacteria recovered in the intestines of the chicks were resistant to the phages.

An interesting strategy was developed for chickens with the aim of amplifying the population of bacteriophages in vivo. The strategy consists of selecting non-pathogenic bacterial hosts that carry the bacteriophage through the gastrointestinal tract and simultaneously allow for the amplification of the phage once in the intestine. Moreover, with the continuous supply of host bacteria instead of phages, the multiplication of the bacteriophage directly into the host could be extended. This strategy may be coupled to the selection of bacteriophages with a wide host range like it was found for two *Salmonella enteritidis* phages, which demonstrated the abilities to infect and multiply in different species of *Salmonella* and in different genera of bacteria, namely *Klebsiella* and *Escherichia* [105]. The establishment of a library of wide-host-range bacteriophages may help mitigate some of the bacteriophage therapy issues.

## 5. Conclusions and Perspectives

Antibiotics are not only crucial tools to cure bacterial infections and successfully prevent infectious complications related to surgical procedures in human and veterinary medicine, but they are also crucial in agriculture to treat plant infections, to preserve and control food-borne pathogens, and to increase productivity in the breeding industry. However, their widespread use and their environmental presence have globally generated intense selective pressure on bacteria, causing a rapid and alarming spread of antibiotic-resistant and MDR bacterial strains. In the present antibiotic resistance era, one of the promising strategies to control bacterial diseases relies on the use of bacteriophages. Phage therapy, which has a long history, has been mostly neglected by the Western world but has re-gained interest as an alternative to conventional antimicrobics in recent decades, especially for diseases lacking vaccines [106].

The effective utilization of bacteriophages as antibacterial agents needs to be supported by a complete understanding and detailed knowledge of the phage itself and through reliable trials according to current regulatory standards. Although a large amount of supportive data are available from past studies involving human patients in Eastern Europe, numerous approaches are being explored. Several clinical trials are in progress to assess the efficacy of both lytic and lysogenic phages in animals and humans. However, the available data are still insufficient to obtain approval from regulatory bodies such as the European Medicines Agency or the US Food and Drug Administration. Lytic phages may cause endotoxicity, while temperate ones need to be modified to act virulent. Moreover, drawbacks such as the emergence of new phage resistance mechanisms, potential interference with endemic phages, refinement of existing anti-phage bacterial defense systems, and the ratio between phages and target bacteria require thorough investigation and elucidation. Ancient work is not being considered because of the lack of safety and because it does not meet the current regulatory standards. The latest progress based on fully regulated randomized clinical trials are more promising to support this approach (Table 1); however, bacteriophages that demonstrate virulence and high efficacy in in vitro studies may not be so effective in in vivo studies. This discrepancy can be attributed to potential patient immune response upon injection, the partial inactivation of phages by the gastric environment after oral administration, or the emergence of phage-resistant mutant bacteria. The tension between in vitro and in vivo results remains a challenge to be solved. Powerful supporting data are provided from animal models, as discussed in this article. Generally, cocktails of mixed phages are more effective than single phages, but the dosage, time(s), and concentration are also important aspects that need to be optimized. More scale-up and commercial trials are envisaged to improve the robustness of this approach, and more translational research is required before phage therapy can be considered clinically feasible on a large scale. Although bacteriophages are increasingly considered crucial in safeguarding both humans and animals in the near future, it is important to acknowledge the major risk of phage therapy, which is the evolution and spread of phage-resistant bacteria. Since antibiotic resistance is a thriving threat, urgent action is required to tackle and bring this pressing issue under control.

## Figures and Tables

**Table 1:** A summary of the in vivo trial studies on bacteriophage therapy in companion and farm animals.

Animal	Target Bacteria	Target Disease	Reference
dog	*Pseudomonas aeruginosa*	chronic otitis externa	[19,20,21]
horse	*Staphylococcus aureus*	superficial pyoderma	[34]
cattle	*Staphylococcus aureus*	mastitis	[39]
heifer	*Escherichia coli*	mastitis	[46]
calf/steer	*Escherichia coli*	diarrhea ^1^	[47,49,50,52,53,54,55,59]
calf	*Escherichia coli*	septicemia	[51]
sheep/lamb	*Escherichia coli*	diarrhea ^1^	[49,55,56,57,58]
pig/piglet	*Escherichia coli*	diarrhea ^1^	[49,63,64]
pig/piglet	*Salmonella enterica*	salmonellosis ^1^	[65,67]
piglet	*Staphylococcus aureus*	nasal infection ^1^	[68]
chicken	*Salmonella enterica* Enteritidis and Typhimurium	salmonellosis ^1^	[73,74,75,76,77,78,79,80,81,82]
chicken	*Salmonella enterica* Gallinarum	fowl typhoid	[83,84]
chicken	*Campylobacter jejuni* and *C. coli*	campylobacteriosis ^1^	[87,88,89,90,91,92,94]
chicken	*Escherichia coli*	septicemia and meningitis	[51]
chicken	*Escherichia coli*	airsacculitis and colibacillosis ^1^	[95,96,97,98,99,100,101,102,103,104]

^1^ Including the shedding of the food-borne zoonotic pathogen.

## Data Availability

Not applicable.
